# Mobile Application-Based Interventions for Chronic Pain Patients: A Systematic Review and Meta-Analysis of Effectiveness

**DOI:** 10.3390/jcm9113557

**Published:** 2020-11-05

**Authors:** Ann-Christin Pfeifer, Riaz Uddin, Paul Schröder-Pfeifer, Felix Holl, Walter Swoboda, Marcus Schiltenwolf

**Affiliations:** 1 Department of Orthopedics, Trauma Surgery and Paraplegiology, Heidelberg University Hospital, Schlierbacher Landstr. 200a, D-69118 Heidelberg, Germany; Marcus.Schiltenwolf@med.uni-heidelberg.de; 2 Institute for Physical Activity and Nutrition (IPAN), Deakin University, Geelong, Victoria 3220, Australia; r.uddin@deakin.edu.au; 3 School of Health and Rehabilitation Sciences, The University of Queensland, St Lucia, Queensland 4072, Australia; 4 Institute of Psychosocial Prevention at the Center for Psychosocial Medicine, University Hospital Heidelberg, Bergheimer Str. 54, D-69115 Heidelberg, Germany; paul.schroeder-pfeifer@med.uni-heidelberg.de; 5 DigiHealth Institute, Neu-Ulm University of Applied Sciences, Wileystr. 1, D-89231 Neu-Ulm, Germany; Felix.Holl@hs-neu-ulm.de (F.H.); Walter.Swoboda@hs-neu-ulm.de (W.S.); 6 Institute for Medical Information Processing, Biometry, and Epidemiology, Ludwig Maximilian University of Munich, Marchioninistr. 15, D-81377 Munich, Germany

**Keywords:** chronic pain, mobile application, rehabilitation, review, meta-analysis

## Abstract

Chronic pain is one of the major causes of disability in the general population. Even though there are effective treatment options available for reducing symptoms, these treatments often do not have consistent lasting effects. As the usage of mobile devices has increased enormously during the last few years, mobile application-based treatment options are widespread. Such app-based programs are not yet empirically proven but might enable patients to become more independent in their pain management in order to prevent relapse. The aim of this meta-analysis was to summarize the literature on mobile application-based interventions for chronic pain patients. Therefore, three electronic bibliographic databases, PubMed, PsycINFO, and Web of Science, were searched for studies that investigated the effectiveness of mobile application-based intervention for chronic pain on pain intensity. The final sample comprised twenty-two studies, with a total of 4679 individuals. Twelve of these twenty-two studies used a randomized control trial (RCT) design, while ten studies only used an observational design. For all twenty-two studies, a small but significant effect (*d* = −0.40) was found when compared to baseline measures or control groups. The results suggest that apps-based treatment can be helpful in reducing pain, especially in the long-term.

## 1. Introduction

Chronic pain, which is defined as “An unpleasant sensory and emotional experience associated with, or resembling that associated with, actual or potential tissue damage that persists over a period of at least three months” by the International Association for the Study of Pain (IASP) 2020, is a significant burden on society [1,2]. Even though the global burden of chronic pain is very high, with prevalence rates between 19% and 37%, the management of the disease is not very effective in the long term [3]. Follow-up studies of chronic pain patients suggest a remission rate of approximately 50% after one year [4] with a non-recovery rate as high as 78% in an extensive cross-national survey for chronic pain [4]. A recent systematic review suggested that about two-thirds of individuals with non-specific lower back pain were still suffering from pain after one year [5]. Even though effective treatment options, such as conservative medical treatment, physical therapy, psychotherapy, or multidisciplinary rehabilitation in primary and specialized care settings, are available for reducing experienced symptoms [6,7,8], these treatments often seem only adequate for a short time with insufficient evidence for long-term success [9].

As the access to and utilization of mobile devices have increased during the last few years, and the technology continuously improves almost daily with new updates and features, a current review of Thurnheer and colleagues indicates that apps for pain management might have some beneficial effects [10]. The majority of the original studies included in that review reported significant improvements in pain over time. The use of an app, therefore, might be useful, particularly in an outpatient setting for the management of pain [10]. Such computer-based programs are cost-effective, easy to implement, and enable patients to become more independent in their pain management in order to prevent relapse [11,12]. Moreover, app-based interventions are accessible almost 24/7 and avoid geographical constraints for people from rural or remote areas [13,14].

Even though apps are becoming more available with advancements of technology [15], only a few app designers included relevant stakeholders such as patients or clinicians in the development process [16], and most of the available apps were not scientifically evaluated before their market release [17,18,19,20]. Previous (systematic) reviews on mobile-based interventions for pain patients mainly investigated the usability and acceptability of apps for acute and chronic pain patients [21]; however, these reviews did not assess the effectiveness and quality of such apps in the management of chronic non-cancer pain.

The aim of this systematic review and meta-analysis was therefore twofold: first, to investigate the efficacy of mobile application-based treatments of chronic non-cancer pain; and second, to rate the quality of the apps in terms of content, ease-of-use, and functionality, from a user point of view. The meta-analytical procedures were applied to estimate the quality of the studies and the efficacy of the utilized apps.

## 2. Materials and Methods

### 2.1. Protocol and Registration

This systematic review and meta-analysis were pre-registered on PROSPERO (Registration number: CRD42019139262). The protocol is available online [22].

### 2.2. Eligibility Criteria

As recommended by the PRISMA guidelines [23], the eligibility criteria were defined according to the PICOS framework: primary studies investigating (sub-) samples of patients suffering from chronic non-cancer pain aged 6–80 years were included. While pediatric chronic pain significantly differs from adult chronic pain [24], only one of the included studies examined patients under the age of 18 years. Consequently, it was not possible to test for differences between studies examining pediatric chronic pain and adult chronic pain. Studies were eligible if they compared mobile application-based interventions with a control group (treatment as usual or another control group) and/or baseline with post-intervention measures. Individuals were classified as chronic pain patients if they suffered from reoccurring pain longer than three months in the primary study. Outcomes were measures of pain intensity. Concerning study designs, between-, within-, and single-group designs were included. Only original research articles were eligible, and case studies, letters to the editor, perspectives, opinions, and reviews were excluded. Published studies in the English and German languages were eligible.

### 2.3. Literature Search

The search strategy was based on the recommendations by Lipsey and Wilson [25]. Three electronic databases (i.e., PubMed, PsycINFO, and Web of Science) were systematically searched up to 30th April 2019. Snowball search method was also applied by screening reference lists of the included articles. The literature search was performed by a trained researcher (ACP) and supervised by a second researcher (FH).

The following key terms were used: chronic pain, pain+, pain management, somatoform pain disorder, non-cancer pain, musculoskeletal pain, fibromyalgia, cellular phone+, mobile devices, smartphone, mobile applications, app, e * health, telehealth, telemedicine+, m * health, mobile health, p * health, and personal health. Database-specific Boolean operators (e.g., AND, OR, NOT) were used. No restriction regarding publication date was applied. The full electronic search strategy is presented in Table S1.

### 2.4. Study Selection

**Figure 1:**
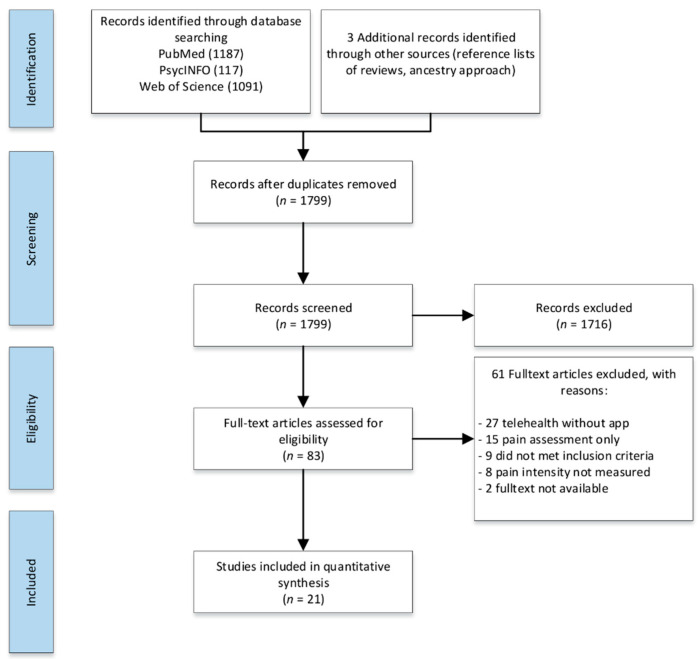
PRISMA flow diagram.

All retrieved articles were imported into EndNote, screened by title and abstract, and duplicate(s) were removed. Studies meeting the eligibility criteria were selected for full-text screening, and eligible studies were identified (Figure 1). If abstracts or full texts or data on the primary outcome or app information were not available, corresponding authors were contacted requesting access to the publication or data. All eligible studies were included in the systematic review, and those providing sufficient data on the outcomes of interest were included in the meta-analysis. Study selection was performed independently by two reviewers (ACP and PSP) with a third reviewer (RU) deciding in case of discrepancies.

### 2.5. Data Extraction

Sample characteristics, including sample size, demographic variables, and nature of possible comparison groups, were extracted. Data on intervention characteristics (i.e., mean pain intensity per group and the number of participants in each group), the duration of symptoms, pain location, diagnostic instrument, and pain intensity were extracted. Data on different aspects of the study design (e.g., randomization, type of control, type of measure) and whether an intervention was evaluated were also extracted.

If mean and standard deviation were not directly reported, they were estimated [26]. If data were not reported in texts or tables but were extractable from figures, an online plot digitizer was used [27]. If relevant data on the outcomes for the meta-analysis were not available, the corresponding authors were requested to provide the required information. Information was extracted in duplicate and independently by two reviewers (ACP and PSP), using a pre-defined data extraction template. In the case of extraction discrepancies, a third reviewer (RU) decided.

### 2.6. Outcomes

The primary outcome of interest of this systematic review was pain intensity. Pain intensity was utilized as the primary outcome of efficacy since alternative outcomes such as the level of functioning or disability were infrequently reported. Efficacy of the apps in terms of pain intensity was operationalized as differences in pain intensity between participants who received the app-based intervention vs. participants who did not receive the intervention. In studies without a control group, differences in pain intensity at the last time point compared to the beginning of the intervention were compared to estimate efficacy. The quality of the apps was assessed with the Mobile App Rating Scale (MARS) [28]. The MARS is a rating instrument for mobile apps and consists of 23 items rated on a 5-point Likert-scale ranging from 1 (inadequate) to 5 (excellent). The items cover the aspects of engagement, functionality, aesthetics, information quality, and subjective quality of the app.

### 2.7. Quality of Studies

For the methodological quality assessment of all included randomized controlled trials (RCTs), four essential criteria were used [29]: selective outcome reporting (reporting bias), adequate sample size, random sequence generation, and incomplete data i.e., intent-to-treat (ITT) analysis. The Cochrane Network risk-of-bias tool for RCTs (RoB) was used to rate the RCTs into four different categories: (1) high risk of bias, (2) unclear risk of bias, (3) low risk of bias, and (4) not applicable. Studies were categorized as low risk if the majority of the key domains were rated with a low risk of bias. For cohort and observational studies with no control group, the Newcastle–Ottawa Quality Assessment Scale (NOS) for cohort or case–control studies [30] was used. Study quality rating was performed in duplicate and independently by two reviewers (ACP and PSP). In the case of rating discrepancies, a third reviewer (RU) decided.

### 2.8. Strategy for Data Synthesis

First, a narrative synthesis of the included studies, summarizing information about their participants, study designs, and primary and secondary outcomes, was conducted. Second, quantitative synthesis of data from individual studies was performed. Hedge’s g was used to summarize differences in the pain intensity between groups or before and after an intervention [31]. If several time points were available, the last one was considered to be the most relevant to the current analysis. *I*^2^ and Q, along with their 95% confidence intervals (CIs), were used as indicators of heterogeneity of the effects reported [31]. Begg and Mazumdar’s rank correlation [32], Egger’s regression test [33], and Duval and Tweedie’s trim-and-fill procedure [34] were applied to test publication bias.

Only studies with comparable rating scales for pain intensity (e.g., Visual Analog Scale (VAS), on a scale of 0–10 or 0–100 and numeric rating scale (NRS), 0–10) were included in the comparative analysis. If necessary, the pain score scales were rescaled to a 0– to 10–point scale.

The results of the meta-analysis are shown in a forest plot. If data were missing and could not be computed from the other available data, corresponding authors were contacted and followed up after two weeks if no response was received. If the authors did not respond, data were considered missing. All analyses were conducted under the random-effects model, using the package meta for R [35].

## 3. Results

A total of 2398 articles were retrieved during the initial search and 1799 articles were identified for the title and abstract screening after removing the duplicates. After title and abstract screening, 83 articles were included in the full-text screening; 22 unique studies [36,37,38,39,40,41,42,43,44,45,46,47,48,49,50,51,52,53,54,55,56,57] with a total of 4679 patients met the eligibility criteria (1515 in non-RCT designs, 3164 in RCT designs) (Figure 1). For the meta-analysis, 12 of these studies were considered as RCTs [36,37,38,39,40,41,42,43,44,45,46,47], while 10 were observational or studies of similar design, which compared baseline measures to post-intervention measures of the same individuals [48,49,50,51,52,53,54,55,56,57]. Studies varied in sample sizes, sex distributions, populations, assessment instruments, and study quality. Six studies were aimed at individuals with general chronic pain [40,41,51,52,56,57], nine studies at individuals with chronic lower back pain (LBP) [36,37,39,44,46,47,49,53,54], three studies at individuals with arthritis (e.g., osteoarthritis and rheumatoid arthritis) [43,45,55], and one study each at individuals with menstrual pain [48], frozen shoulder pain [38], chronic neck pain [42], and migraine [50]. Fifteen of the studies recruited patients during clinic visits or through their general practitioners (GP) [36,37,38,40,41,42,43,46,47,48,51,52,55,56,57] or research institutions, and seven recruited participants from the community via the internet or flyers [39,44,45,49,50,53]. All studies included both sexes, except two which included only women [41,55]. The app-based interventions were delivered via a smartphone or tablet and lasted between 4 weeks and 12 weeks. Most of the studies were conducted in the USA (*n* = 8) [39,40,43,44,45,52,55,57], followed by Germany (*n* = 5) [46,48,49,50,53]. In most studies, the majority of the patients were females, married, and had a mean age between 23.7 (SD = 3.9) and 68.52 (SD = 7.65) years. A total of twenty apps for the treatment of pain were examined. Selected characteristics of the included studies are presented in Table 1.

In all studies, only one measurement was utilized to calculate standardized mean differences. Nineteen studies used a visual analog or numeric rating scales of pain as outcome measures, one study each used the Arthritis Impact Measurement Scale 2 (AMS2), the brief pain inventory (BPI), and the Patient-Reported Outcomes Measurement Information System (PROMIS) (PROMIS) (see Table 1).

In the current analyses, ten observational studies investigated the effect of pain apps [48,49,50,51,52,53,54,55,56,57]. All apps included pain tracking tools; one used Fitbit for the tracking of physical activity [58]. Additionally, most apps also offered self-management options for pain. Content-wise, the examined apps utilized a variety of interventions. One of the studies used an app with instructions for self-acupressure [59], one included an optical imaging tool [55], one a digital music intervention [51], one daily reminders along with supportive messages [52], two utilized a mix of app-guided physiotherapy exercises, mindfulness, and education [46,49,53], one employed self-help chats moderated by experts [50], and one app had a medication management option [56].

Of the 12 RCTs, only one compared their app-based intervention with an assessment only group [43]. The other 11 RCTs compared their app-based intervention with active control groups that received either physiotherapy [46,47], educational reading material or other such information [36,40,42,44], recommendations to stay active [37,38], access to a self-help website [41,59], a wearable activity tracker without smartphone application [45], or unspecified treatment as usual with mail reminders to complete assessments [39]. A table with a detailed description of the app content can be found in the Supplementary file (See in Table S2).

**Figure 2:**
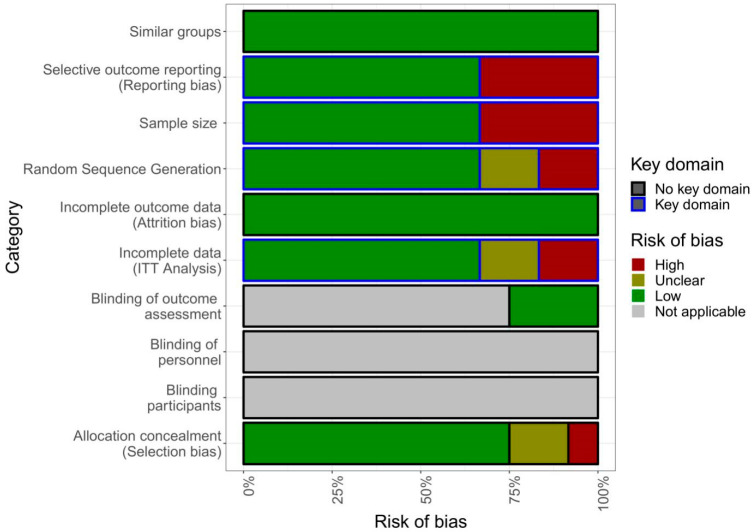
Risk of bias analysis. ITT, intent-to-treat.

The quality of studies included in the meta-analysis was not optimal. Only eight met at least three of the four pre-defined key domains of the quality criteria, namely random sequence generation (selection bias), incomplete data (ITT analysis), selective outcome reporting (reporting bias), and adequate sample size (see Figure 2) [36,37,38,39,40,41,43,44].

While the Cochrane Network recommends blinding of participants as a key domain, particularly for pharmaceutical studies regarding pain [29], this is not applicable for alternative intervention studies such as the ones included in this systematic review, since the participants cannot be blinded towards receiving treatment. Most studies did not specify treatment duration since most apps were made available to the participants for as long as the participants wanted to use the app, instead of fixed treatment duration, as would be the case for most offline treatments for chronic pain. As such, dosage effects are difficult to estimate.

**Figure 3:**
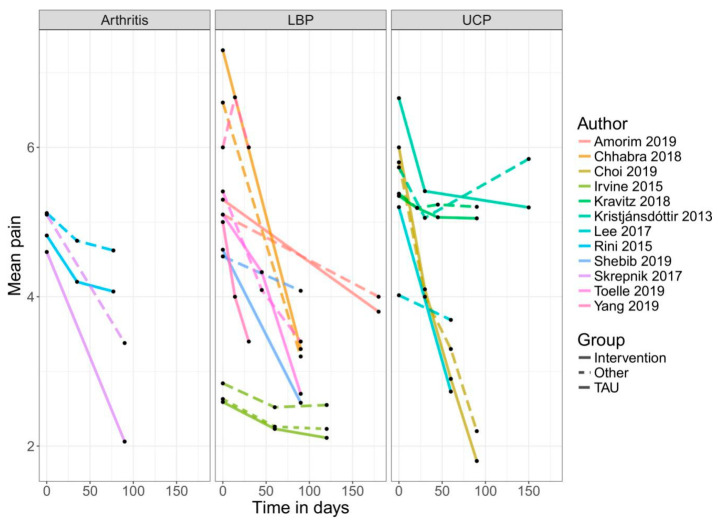
Improvement in pain over time in RCTs. Abbreviations: LBP = low back pain; UCP = unspecific chronic pain; TAU = treatment as usual.

**Figure 4:**
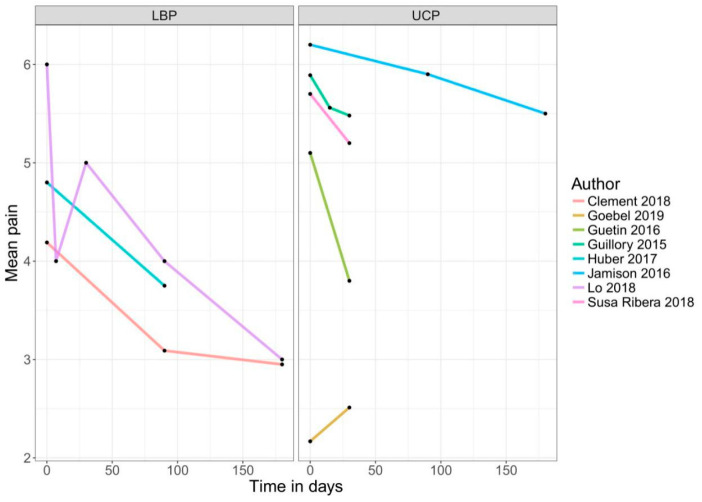
Improvement in pain over time in pre–post studies. Abbreviations: LBP = low back pain; UCP = unspecific chronic pain.

**Figure 5:**
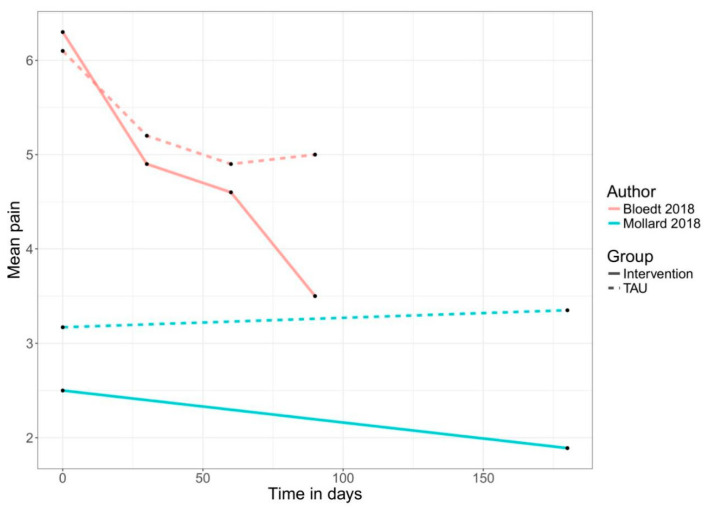
Improvement in pain over time in group comparison. Abbreviations: TAU = treatment as usual.

An app-based intervention of pain was compared with a control group (treatment-as-usual, alternative treatment, non-specific control, or waiting list) or baseline measures of the same individuals in 22 comparisons. Two of the included RCTs compared an app-based intervention with a co-intervention, such as text message support or self-acupressure, with the app only intervention [48,52]. Therefore, only the intervention arms of these RCTs were used in the meta-analysis in the non-RCT section of this meta-analysis. Another RCT used a three-arm design comparing the app-based treatment with a control group and an alternative treatment [39]. For the analysis in the present review, the intervention versus control arm was used because of a lack of description for the alternative treatment. Figure 3, Figure 4 and Figure 5 show the mean trajectory of the respective outcomes for all studies over time.

**Figure 6:**
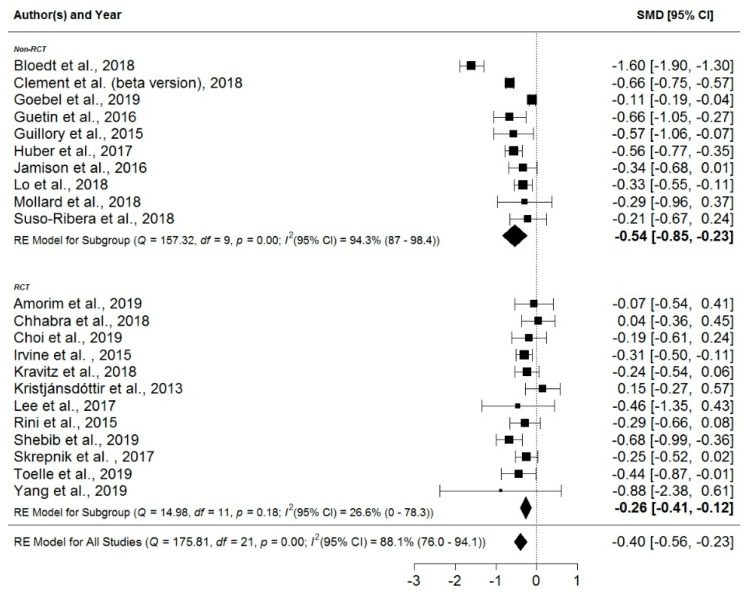
Forest plot. Abbreviations: CI = confidence interval; *df* = degrees of freedom; RCT = randomized-controlled trial; RE = random effect; SMD = standardized mean difference.

**Figure 7:**
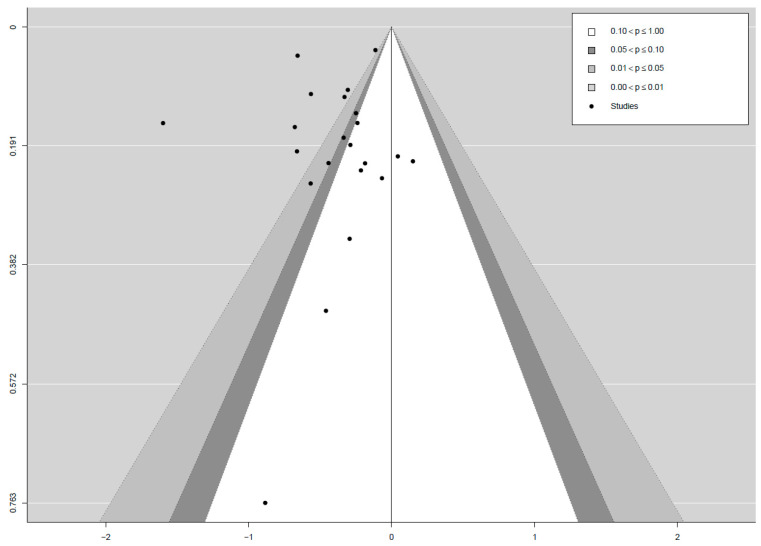
Funnel plot.

The mean effect size was *d* = −0.4 (95% CI: −0.56, −0.23). Heterogeneity was high at *I*^2^ = 88% (95% CI: 76.0, 94.1). Because of the small number of studies, only one subgroup analysis for RCT vs. non-RCT designs was conducted. When limiting the analysis to the 12 studies with an RCT design, a smaller effect of *d* = −0.26 (95% CI: −0.41, −0.12) was found, albeit with a much lower heterogeneity of *I*^2^ = 26.6% (95% CI: 0.0, 78.3). Because of the small number of studies, however, the associated 95% CIs are wide, ranging from no heterogeneity to high heterogeneity. In nine out of the ten studies with non-RCT designs, the effect sizes, based on the improvement in pain from baseline to the primary endpoint of the respective studies, were computed to get an impression of the improvement participants made using the pain apps compared to baseline measures. While these effects do not indicate an effect of the examined pain apps per se, as the randomized nature of an RCT is lacking, they nevertheless might provide a conservative estimate of the general feasibility of these interventions. For the subgroup of non-RCT design studies, a larger effect of *d* = −0.54 (95% CI: −0.85, −0.23) was found, coupled with a much larger heterogeneity of *I*^2^ = 94% (95% CI: 87.0, 98.4). The corresponding forest plot summarizing the effect sizes of the different studies, subgroups, and their 95% CIs can be found in Figure 6. Both a contoured funnel plot (see Figure 7) and Egger’s regression test (*t* = −0.07, *df* = 20, *p* = 0.94), using the standard error as predictor, did not indicate publication bias.

### Assessment of Quality of the Apps

Only five apps were available on Google Android Market or Apple App store. Most of these apps—except one—were either not freely accessible to users or geo-locked (only available to users in a specific country or region). We requested access to the apps from their corresponding authors and received feedback from two authors, of whom one offered a pdf layout of the app as the original app was no longer in use. In order to rate an app, MARS requires that the rater experience and interact with the app firsthand by using it. As we did not have access to the majority of the apps to use or interact with, we decided not to rate the apps.

## 4. Discussion

The aim of this systematic review and meta-analysis was to summarize the literature on mobile application-based treatments for non-cancer chronic pain patients and to examine the efficacy as well as the quality of the utilized apps. Twenty-two unique studies of individuals utilizing apps for the treatment of different forms of pain were examined in this meta-analysis.

### 4.1. Efficacy of Mobile Application-Based Treatments

A small but significant effect (*d* = −0.40) was found when compared to baseline measures or control groups. In RCTs, when apps for the treatment of pain were directly compared to control groups, it was found that the pain apps were significantly more effective in reducing pain with a small effect size (*d* = −0.26). When comparing baseline measures of pain with post-intervention measures of pain in studies, which did not employ an RCT design, a small to a medium reduction in pain was found (*d* = −0.54). However, these effects should be interpreted with caution as most of the interventions used co-interventions such as supportive text messages or phone calls, activity tracking tools, and self-management booklets in addition to mobile apps. One study used motivational interviewing for the intervention group before using the app but not for the control group, which might have had an impact on the intervention effect of the app [43]. It is, therefore, possible that the effects were not exclusive to the mobile app used, and other intervention components supplemented the effects. Since these additional components were only offered to the intervention group(s), and not to the control groups, we cannot determine whether or not the app, the additional or co-intervention, or a combination of both, led to the final effect. Other meta-analyses showed that computerized interventions for depression were more effective when additional personal support was offered compared to interventions without support [60,61], which might bolster the latter hypothesis.

The included studies were heterogeneous in terms of the investigated chronic pain conditions as well as in terms of the examined populations. Not only were different chronic pain patients targeted, such as unspecific (e.g., low back pain) and specific (e.g., arthritis) pain patients, but the studies also used different definitions of chronic pain. While some studies defined chronic pain as pain that lasts for at least six weeks [40,44,46], other studies used the general definition and more conservative definition of more than 3–6 months [1,2,62].

### 4.2. Quality of the Application-Based Treatments

Though we planned to assess the quality of the apps (or rate the apps) used in the included studies, we were not able to because all but two of the apps were unavailable. Upon contacting the authors, the primary reason for this was that the apps were hosted on study servers for the duration of the studies only and not freely accessible via platforms such as Google Play or Apple App Store. Since the assessment of app quality using the MARS [28], which is widely used to rate apps in academic studies, requires the rater to use and experience the app firsthand, we were unable to apply the instrument.

### 4.3. Comparison with Existing Literature

To the best of our knowledge, this is the first meta-analytic review investigating the effectiveness of smartphone or tablet apps in the treatment of non-cancer chronic pain. A similar review by Thurnheer and colleagues (2018), which assessed the efficacy of apps in the management of pain for both cancer and non-cancer pain, concluded that out of the fifteen included studies a majority reported beneficial effects of the apps on pain [10]. While Thurnheer and colleagues did not attempt a quantitative synthesis because of the high heterogeneity between the included studies, the findings of the present study corroborate Thurnheer’s findings.

With regard to the quality of studies assessing apps for pain, the present study confirms the findings of other authors. Several reviews have criticized the available apps and corresponding studies, both in terms of content validity, e.g., [17,20], and scientific standards [19]. Additionally, a common flaw in the development of apps for pain self-management is that neither healthcare professionals such as medical doctors, psychologists, or physiotherapists, nor patients, are involved in the process [17,63]. The present study is consistent with the findings of Machado and colleagues [18], reporting that most of the available apps being assessed lacked an empirical underpinning and, while generally, interventions that are endorsed by guidelines are employed, the implementation quality is often low.

Concerning the effects of apps for the treatment of other conditions, the present study also corroborates the findings in the literature. Apps have been shown a similar positive effect on several psychological (e.g., anxiety or depression) [64,65], as well as physiological symptoms (e.g., asthma) [66], or adherence to diabetes treatment [67]. Judging by other studies on chronic conditions, the inclusion of motivational elements might be beneficial, especially for elderly users [67].

### 4.4. Limitations

There are several methodological limitations concerning the selected studies. First, similar to internet-delivered and computerized interventions, app-based interventions suffer from a lack of reliable diagnostic instruments [68]. In the present study, this is amplified by the highly subjective and multifaceted nature of pain as the main outcome [69]. While single-item measures such as VAS and NRS are widely used, as is the case with the selected studies in this review, they do not capture the multifaceted nature of pain. Additionally, these instruments are often administered only once for a given measurement point instead of multiple times, such as with ecological momentary assessment designs, which raises the question of their reliability. While some studies did utilize more comprehensive measures of pain, such as the BPI, they were in the stark minority.

A second methodological shortcoming that affected all of the RCTs included in this study is the insufficient sample size. Tashjian and colleagues found a rough estimate for the minimally significant difference in pain, measured on a VAS from the patients’ perspective, to be *d* = 0.51 [70]. In order to find such a difference in a two-sided *t*-test indicating the difference between two independent means at a power of 0.9 and an α level of 5%, the sample size needed, calculated by the software G * Power (Heinrich Heine University Düsseldorf, Düsseldorf, Germany), would be 164 total or 82 per group. The difference of *d* = 0.51 represents a comparatively big minimally significant difference in comparison to other conditions such as depression, and is thus relatively easy to find, requiring only medium sample sizes. Nevertheless, applying these standards, only four out of the twelve RCTs [39,40,44,45] included in this study had sufficient power to find such a minimally significant difference.

Apart from the limitations of the included studies, this meta-analysis also has some limitations. While heterogeneous treatments and samples were included, it was not possible to include sufficient studies to be able to investigate the differences between studies via sub-sample analyses and meta-regressions. In addition, only studies published in English or German were included. However, a comprehensive, unspecific search strategy was applied in multiple databases in order to include all relevant studies. The lack of studies thus seems to be symptomatic for the field of research in the English or German language.

With digital technologies gaining popularity in health research, more apps are being designed and used in different interventions. Going forward, a repository of mobile apps for research purposes would be required to ensure that apps are freely available to be tested and rated.

Going forward, more methodologically sound studies on the efficacy of mobile application-based interventions of chronic non-cancer pain are needed. These studies should focus on more reliable outcome measures or alternative, more informative outcomes of intervention such as level of functioning, assessed in appropriately sized samples. Additionally, these studies should make their apps available to the scientific community so that rigorous quality testing can be done, an aspect of mobile application-based treatments that is sorely lacking at the moment.

## 5. Conclusions

Despite these limitations, our study demonstrates emerging evidence that mobile apps can be useful in reducing pain among non-cancer pain patients. As electronic health and mobile health continue to evolve, more research with robust methodologies and well-designed apps is required to understand how to utilize this digital technology best to help patients with pain. More studies are needed to investigate which programs work and for which population. Future research should also focus on how con-interventions or additional intervention components may affect the utility of pain apps.

## Figures and Tables

**Table 1:** Study characteristics.

First Author, Year	Type of Study	Target Population	% Female	Recruitment	Inclusion	N	Intervention	Additional Support	Intervention Duration	Primary Endpoint	Outcome Measure	Country
**Amorim 2019**	RCT	Adults (18–65 years)	50%	via clinic	Chronic low back pain: - mechanical LBP for over 12 weeks	68	1. Intervention group: Mobile web app; 2. Control group: Information booklet and staying active	YES: After the first face-to-face coaching session, the health coach contacted participants fortnightly and information booklet + Fitbit tracker	not specified	6 months	Pain NRS	Australia
**Bloedt 2018**	Randomized pragmatic trial (observational study)	Women (18–34 years)	100%	via research institution	Menstrual pain (cramping): - being diagnosed with dysmenorrhea	221	1. Intervention group: AKUD App with acupressure features	No: Usual care	not specified	6 months (6 menstrual cycles)	Pain NRS	Germany
**Chhabra 2018**	RCT	Adults (>18 years)	n/a	via clinic	Chronic low back pain: - mechanical LBP >12 weeks with or without radicular symptoms	93	1. Intervention group: Snapcare App; 2. Control group: Usual care with written prescription of medication and physical activity	No: Usual care (Written prescription of medication and physical activity)	12 weeks	12 weeks	Pain NRS	India
**Choi 2019**	RCT	Adults (>20 years)	68%	via clinic	Frozen shoulder: - shoulder pain for at least one month	84	1. Intervention group: Exercise app, including feedback, motivation, reminder; 2. Control group: Self-exercise group	YES: both groups were prescribed nonsteroidal anti-inflammatory drugs (celecoxib) for two months, and educated and encouraged to perform self-exercise	not specified	12 weeks	Pain VAS	Korea
**Clement 2018**	retrospective analysis of the user database	Adults (>18 years)	49%	via online channels (Facebook, Google Ads, company home page)	Low back pain: - declaration of medical treatment of back pain	1055	1. Intervention group: Updated 1.4 version of the Kaia App featuring physiotherapy, mindfulness, and education	No	not specified	24 weeks	Pain NRS	Germany
**Goebel 2019**	Observational study	Adults (age not reported)	87%	via online channels (clinic website, social media, newsletters)	Migraine: - suffering from migraine or headaches	1464	1. Intervention group: Migraine app with medication reminder, expert chats, relaxation, education, couching	No	not specified	max. 12 months (no primary endpoint defined)	Pain VAS	Germany
**Guetin 2016**	Observational study	Patients (7–88 years)	79%	via clinic	Different chronic pain conditions	53	1. Intervention group: Music-care app receptive music intervention (max. 7 sessions)	No	not specified	After use of app (min. 1 session and max. 7 sessions)	Pain VAS	France
**Guillory 2015**	Pilot RCT (Observational study)	Adults (18–80 years)	75%	Via clinic	Chronic non-cancer pain: - pain on most days for >3 months	82	1. Intervention group: Pain tracking app usage + twice daily text messages reminder	YES- daily reminder to use the app plus twice-daily supportive text messages for encouragement	4 weeks	4 weeks	Pain NRS	United States
**Huber 2017**	retrospective study	Adults (mean age of 33.9)	58%	via online channels (FB, Google ads, company homepage)	Unspecific low back pain: - declaration of medical treatment of back pain	180	1. Intervention group: Kaia mobile app that digitalizes multidisciplinary pain treatment	NO	not specified	12 weeks	Pain NRS	Germany
**Irvine 2015**	RCT (Comparison App vs. Control)	Adults (18–65 years)	60%	via online channels (FB, Google ads, company homepage)	Non-specific low back pain: - low back pain within the past 3 months	597	1. Intervention group: FitBack app; 2. Control group: Usual care with reminder E-mails; 3. Alternative care group: 8 E-mails with link to resources	YES: Weekly E-Mail reminder	not specified	16 weeks	Pain intensity (1–7)	United States
**Jamison 2016**	Observational study	Adults (>18 years)	64%	via clinic	chronic pain: - chronic pain for >6 months	90	1. Intervention group: Pain coping app + Fitbit	No: only technical support was offered	12 weeks	12 weeks	Brief pain inventory (BPI) -> Pain intensity (0–10)	United States
**Kravitz 2018**	RCT	Adults (18–75 years)	47%	via research institutions	CMSP: - musculo-skeletal pain for >6 weeks at the time of screening	215	1. Intervention group: Mobile health app (choice of e.g., drug or alternative treatments); 2. Control group: TAU + self-management booklet	YES: Reminder phone calls or e-mail + self-management booklet	not specified	48 weeks	Pain intensity (PROMIS 3a short form) (0–100)	United States
**Kristjánsdóttir 2013**	RCT	Women (>18 years)	100%	via clinic	CWP: - having suffered from CWP for more than 6 months	140	1. Intervention group: Smartphone intervention with diaries and daily feedback; 2. Control group: Informational website with self-help material	YES: Access to an informational website with self-help pain-management material	4 weeks	4 weeks	Pain VAS	Norway
**Lee 2017**	RCT	Adult office worker (25–35 years)	45%	via research intuition	Chronic neck pain: - pain for more than 6 months	20	1. Intervention group: App with self-feedback for exercises; 2. Control group: Brochure and one education session on care their neck pain	YES: Both groups received text messages once a week about caring for their pain	not specified	8 weeks	Pain VAS	Korea
**Lo 2018**	Observational study	Adults (18–65 years)	25%	via homepage invitation of clinic	Chronic neck and back pain: - pain within the past 3 months	161	1. Intervention group: Artificial intelligence (AI) embedded smartphone app	No: But contact function via in-app messaging function	not specified	4 weeks	Pain NRS	China
**Mollard 2018**	Pilot study two group experimental design	Adults (>18 years)	n/a	via clinic	rheumatoid arthritis (RA): - actively seeing a rheumatology provider at the researchers’ university rheumatology clinic	36	1. Intervention group: Live with Arthritis app to monitor progression of rheumatoid arthritis inflammation using optical imaging; 2. Control group: TAU	No	not specified	6 months	Pain VAS	United States
**Rini 2015**	RCT	Adults (>18 years)	81%	via research institution	Osteoarthritis pain: - confirmed radiographically (Kellgren & Lawrence grade ≥ 2, with pain in the affected joint); - Osteoarthritis pain pain > 3 months	113	1. Intervention group: PainCOACH app including coping skills training, guided instructions, individualized feedback, interactive feedback and demonstrations; 2. Control group: Assessment only	YES: Brief regular phone calls phoned to encourage continued use of the program	11 weeks	11 weeks	Pain (AIMS2) ->pain in the prior month (1 = severe –5 = none)	United States
**Shebib 2019**	RCT	Adults (>18 years)	41%	via participating employers across 12 locations in the US	Unspecific low back pain: - pain for at least 6 weeks in the past 12 months	177	1. Intervention group: App including personal coaching in a team to provide peer support; 2. Control group: Three digital education articles from the intervention + TAU	YES: Intervention participants received a tablet and two Bluetooth wearable motion-sensors to be placed along the lower back and torso during the in-app exercise therapy + TAU	12 weeks	12 weeks	Pain VAS	United States
**Skrepnik 2017**	RCT	Adults (30–80 years)	50%	via selected private community-based practices	Knee Osteoarthritis: - knee OA whom the physician investigator decided to treat with one 6-mL injection of hylan G-F 20	211	1. Intervention group: App “OA GO” including motivational messages, pain and mood tracking; 2. Control group: regular follow-up + wearable activity monitor	YES: Regular follow-ups as per standard-of-care following Hylan G-F 20 treatment + wearable activity monitor	90 days	90 days	Pain NRS	United States
**Suso-Ribera 2018**	Feasibility Study	Adults (18–65 years)	53%	via clinic	Heterogenous chronic pain: - pain for more than 6 months prior to the study	38	1. Intervention group: Ecological momentary assessment (EMA) monitoring app with protocol for pain, mood and medication (e.g., side effects)	YES: Weekly phone calls to assess recalled pain intensity and mood	30 days	30 days	Brief Pain Inventory (BPI) -> Pain NRS	Spain
**Toelle 2019**	RCT	Adults (18–65 years)	70%	via clinic	Unspecific low back pain: - non-specific low back pain; - pain had to be ongoing for the last 6 weeks up to 12 months	101	1. Intervention group: Kaia App including modules: (1) education, (2) physiotherapy, and (3) relaxation; 2. Control group: Six face-to-face sessions of standard physiotherapy once a week + weekly E-mails with online resources	No	12 weeks	12 weeks	Pain NRS	Germany
**Yang 2019**	RCT	Adults (>18 years)	50%	via clinic	Chronic low back pain: - confirmed diagnosis of chronic low back pain (>3 months) by physicians; - no musculo-skeletal origins	8	1. Intervention group: Self- management app (Pain Care); 2. Control group: Physiotherapy	YES: Physiotherapy	4 weeks	4 weeks	Pain VAS	China

Abbreviations: NRS = Numeric Rating Scale; RCT = randomized controlled trial; VAS = Visual Analog Scale; OA = osteoarthritis; TAU = treatment as usual; AKUD= acupressure against dysmenorrhea; LBP = low back pain; FB = Facebook; PROMIS = Patient-Reported Outcomes Measurement Information System; n/a = data not available; CMSP = chronic musculoskeletal pain; CWP = chronic widespread pain.

## Supplementary Materials

The following are available online at https://www.mdpi.com/2077-0383/9/11/3557/s1, Table S1: Search string, Table S2: App content.
