# Supplementary material for: Mobile Application-Based Interventions for Chronic Pain Patients: A Systematic Review and Meta-Analysis of Effectiveness
Source: J Clin Med. 2020 Nov 5;9(11):3557. doi: 10.3390/jcm9113557 (PMC7694405; doi:10.3390/jcm9113557)
Supplement: Supplementary file 1 [file jcm-09-03557-s001.pdf]

Table S1. Search string.

Database	Boolean Combinations Applied	References Identified
PubMed	(Pain [MeSH Terms] OR Chronic pain [MeSH Terms] OR Pain management [MeSH Terms] OR Chronic pain [Title/Abstract] OR pain * [Title/Abstract] OR non cancer pain [Title/Abstract] OR pain management [Title/Abstract] OR somatoform disorder [Title/Abstract] OR musculoskeletal pain [Title/Abstract] OR fibromyalgia [Title/Abstract]) AND (cellular phone * [Title/Abstract] OR mobile devices [Title/Abstract] OR smartphone [Title/Abstract] OR smart * phone [Title/Abstract] OR mobile applications [Title/Abstract] OR app [Title/Abstract] OR e * health [Title/Abstract] OR n * health [Title/Abstract] OR m * health [Title/Abstract] OR mobile * health [Title/Abstract] OR telehealth [Title/Abstract] OR tele * rehabilitation [Title/Abstract] OR telemedicine * [Title/Abstract] OR telemedicine [MeSH Terms] OR phealth [Title/Abstract] OR personal health [Title/Abstract])	1187
Web of Science	(TS = (Chronic pain) OR TS = (pain+) OR TS = (non cancer pain) OR TS = (pain management) OR TS = (somatoform disorder) OR TS = (musculoskeletal pain) OR TS = (fibromyalgia) NOT TS = (cancer) NOT TS = (cancer pain))<br>AND<br>(TS = (cellular phone) OR TS = (mobile devices) OR TS = (mobile application) OR TS = (app) OR TS = (e * health) OR TS = (n * health) OR TS = (m * health) OR TS = (“mobile health”) OR TS = (telerehabilitation) OR TS = (telehealth) OR TS = (telemedicine) OR TS = (“personal health”) OR TS = (p * health))	1091
PsycINFO	AB ((chronic pain) OR (pain+) OR (non cancer pain) OR (pain management) OR (somatoform disorder) OR (musculoskeletal pain) OR (fibromyalgia)) AND AB ((“cellular Phone”) OR (“mobile devices”) OR (“smartphone”) OR (“mobile applications”) OR (“mobile application”) OR (“app”) OR (“apps”) OR (“ehealth”) OR (“nhealth”) OR (“mhealth”) OR (“e-health”) OR (“n-health”) OR (“m-health”) OR (“mobile health”) OR (“telehealth”) OR (“telemedicine”))	117

Table 2. App content.

Author, Year	Name of App	Intervention	App Content
Amorim_2019	IMPACT app	First face-to-face session lasting 1–2 h, including motivational interviewing, then individually tailored physical activity plan with guidance of health coach, fortnightly check-up calls (in total 12 calls over 6 months), received app with fitbit activity tracker + received a physical activity and sedentary behavior information booklet developed by the Australian Government Department of Health called "Make your move—Sit less, be active for life"	Mobile web app IMPACT to monitor goals, physical activity and related goals (app information used for phone sessions).
Bloedt_2018	AKUD	Only AKUD app with acupressure features	AKUD included a visualization of the menstrual cycle, questionnaires, and diaries for both groups. Acupressure specific features were available only for the acupressure group. These included explanations of the acupressure procedure, drawings, videos, and photos of the acupressure points, as well as a timer to guide the 1-min acupressure of each point.
Chhabra_2018	Snapcare App	Snapcare App + written prescription of medication and physical activity + treatment as usual	App snapcare included daily activity goals, progress, motivation. The goals were set so a gradual increase in physical activity is possible. The goals included aerobic exercises (walking/running), and a set customized home exercises.
Choi_2019	N/A	Participants were prescribed the nonsteroidal anti-inflammatory drugs (celecoxib) for two months, and educated and encouraged to perform a self-exercise program and received a smartphone assisted exercise program	Smartphone application provides three kinds of exercises—forward flexion, cross-body adduction, and sleeper stretch exercises—and its simple user interface, which included real-time visual and auditory feedback, was designed to give patients the motivation to exercise plus an alarm function as a reminder.
Clement_2018; Huber_2017; Toelle_2019	Kaia app	Only Kaia app	The Kaia App involves three therapy modules: (1) back pain-specific education, (2) physiotherapy/physical exercise, and (3) mindfulness and relaxation techniques. Daily content consists of all three therapy modules. The updated content of the 1.4 version of the Kaia App features an increased pool of each of the different exercise

			types (physiotherapy, mindfulness, and education).
Goebel_2019	Migräne-App	Only Migräne-App	Migraine app with documentation function, medication reminder data, expert chats, relaxation, education, coaching.
Guetin_2016	Music Care	Music Care app receptive music intervention (max. 7 sessions)	Music Care application is a receptive music intervention, allowing the patient to listen to a standardized musical sequence of 20 min in length. All the music is specially composed and recorded for the application. Each standardized music session of 20 min is broken down into several phases.
Guillory_2015	Tracking app	Pain tracking app with daily reminder to use the app + twice daily supportive text messages for encouragement (28 messages) that were crafted based on dimensions of social support from an established taxonomy of social support: emotional support (e.g., “You are a strong and courageous person.”)	Ecological momentary assessment (EMA) to track pain variables via diary methods and help users to cope with and manage the pain.
Irvine_2015	FitBack	FitBack with weekly E-Mail reminder. Users receive weekly emails with gain-framed pain self-care messages and prompts to return to the FitBack program to track pain and self-care activities.	FitBack is designed to encourage users to adopt appropriate pain prevention behaviors, tracking progress. Users are constantly encouraged to report their current level of back pain using a 10-point “pain dial” adapted from the Wong Baker pain scale. Users also track their daily pain management activities using an “activity picker” populated with pain self-care activities in four categories (rest and relief, mindfulness, general fitness, and back pain-specific stretching and strength exercises) developed with the panel of pain experts and physicians.
Jamison_2016	N/A	Pain coping app + fitbit + technical support for the users	Components of the smartphone application included: (1) demographic and contact information, (2) comprehensive baseline chronic pain assessment with body map, (3) daily assessments with push notification reminders, (4) personalized goal setting (e.g., exercise routine, weight management), (5) topics of interest with psychological and medical management strategies (e.g., Gate Control Theory, stress and relaxation, managing sleep disturbances, weight management

			and nutrition, problem solving strategies), (6) self-affirming positive statements, (7) saved progress line graphs directed to the health care provider, and (8) past summary logs.
Kravitz_2018	N/A	Mobile health app (choice of e.g., drug or alternative treatments) + reminder phone calls or email + self-management booklet	Users selected from 8 treatment categories: (1) acetaminophen;(2) any nonsteroidal anti-inflammatory drug; (3) acetaminophen/codeine; (4) acetaminophen/hydrocodone; (5) acetaminophen/oxycodone; (6) tramadol; (7) complementary/alternative treatments such as massage, meditation, or physical exercise; or (8) current ongoing therapy (or no therapy).
Kristjánsdóttir _2013	N/A	Smartphone intervention with diaries and daily feedback + access to an informational website with self-help pain-management material	The key ACT concepts included four components: 1. Face-to-face Session, 2. Web-based diaries, 3. written situational feedback, 4. Audio files with guided mindfulness exercises.
Lee_2017	N/A	Besides the app, participants also received text messages once a week about caring for their pain	App with self-feedback for exercises and to track their pain process. The feedback function for participants allows them to observe their accumulative exercise records and the changes in pain themselves by checking the current pain and time immediately after each exercise.
Lo_2018	Well Health	App only	Artificial intelligence (AI) embedded smartphone app including the assessment method, the exercise programs, and exercise instructions.
Mollard_2018	LiveWith Arthritis app	App only	LiveWith Arthritis app is a mobile app that allows users to monitor the progression of RA inflammation and deformity in their hands using optical imaging from a mobile device camera. It also includes self-management behaviors to monitor and manage their RA by tracking pain levels, treatments, and other lifestyle and environmental data (e.g., diet, activity, and weather). Additionally, reports for doctors can be subtracted and helps them provide better care.
Rini_2015	PainCOACH	App + brief regular phone calls to encourage continued use of the program	PainCOACH app including coping skills training, guided instructions, individualized feedback, interactive feedback and animated demonstrations. The program included eight modules without therapist contact. One module per week was recommended and took 35–45 min to complete and provided

			interactive training in cognitive or behavioral pain coping skills.
Shebib_2019	N/A	Participants received a tablet with the app installed and two bluetooth wearable motion-sensors to be placed along the lower back and torso during the in-app exercise therapy + TAU	App with digital care program for LBP including personal coaching in a team to provide peer support through a discussion feed within the app.
Skrepnik_2017	OA GO	Regular follow-ups as per standard-of-care following Hylan G-F 20 treatment + wearable activity monitor	App "OA GO" including motivational messages, pain and mood tracking and requested that the patient enter pain and mood data on a once-daily basis.
Suso-Ribera_2018	Pain Monitor	App + weekly phone calls to assess recalled pain intensity and mood	Ecological momentary assessment (EMA) monitoring app with protocol for pain intensity, use of rescue analgesics, side effects of the medication, interference of pain in functioning (sleep, work, leisure, and social interactions), fatigue, mood (happiness, depression, anxiety, and anger), perceived health status, and activity level, as well as psychological and behavioral factors that influence the pain experience.
Yang_2019	Pain Care	App + physiotherapy	Self- management app (Pain Care) consists of three elements: (1) New pain episode; (2) View data, (3) About and tools. Users put in pain intensity and activity levels before and after each session. They can also view and print out their data and share it with the assessor. The reminder/alarm functions can also be tailored to the users' needs.

N/A = data not available.
